# “Are we still at this point?:” Persistent misconceptions about the adequacy, rigor and quality of qualitative health research

**DOI:** 10.3389/fpubh.2025.1586414

**Published:** 2025-04-22

**Authors:** Ana Moura, Sílvia Fraga, Pedro D. Ferreira, Mariana Amorim

**Affiliations:** ^1^EPIUnit ITR, Institute of Public Health of the University of Porto, University of Porto, Porto, Portugal; ^2^Centre for Research and Intervention in Education (CIIE), Faculty of Psychology and Education Sciences, University of Porto, Porto, Portugal

**Keywords:** qualitative research, health sciences, methodology, ethics, teaching, artificial intelligence

## 1 Introduction

As researchers and teachers experienced in quantitative, qualitative, and mixed methods, we have witnessed persistent misconceptions about qualitative research (QR) in health-related fields. While quantitative methods are often considered the gold standard, QR remains undervalued and is perceived as less rigorous. This skepticism stems from applying quantitative criteria—such as generalizability and statistical significance—to QR, disregarding its focus on depth, context, and reflexivity. QR enables understanding complex health phenomena, capturing lived experiences, and informing context-sensitive policies. For instance, QR has been offering a more holistic understanding of barriers to healthcare access, patient adherence to treatments, and the impact of chronic illnesses on daily life. An illustrative example of qualitative research's institutional relevance can be found in regulatory settings, such as the Food and Drug Administration's Patient-Focused Drug Development initiative. This framework explicitly integrates qualitative methods to better understand patient experiences, identify treatment priorities, and develop patient-reported outcome measures in drug development. Such mandates reflect the indispensable role of qualitative insights in developing meaningful and patient-centered healthcare innovations.

Despite their critical role, QR is frequently marginalized, which is particularly problematic in public health, where qualitative insights help uncover structural health determinants essential for designing tailored policies, interventions, and practices. Addressing these misconceptions requires an integrative perspective that values each methodology's unique contributions to evidence-based decision-making.

Notwithstanding the dominance of positivist traditions, there is a growing recognition of qualitative methodologies in competitive funding calls and research agendas. The increasing emphasis on participatory, interdisciplinary and innovative approaches has opened space for more diverse methodological approaches and positioned qualitative methods as tools for delivering comprehensive and efficient responses to health issues. However, this growing visibility—especially in the context of rapid digital transformation—necessitates a careful and reflexive evaluation of their application to ensure their responsible, rigorous, and ethical use.

This opinion article contributes to the debate by addressing enduring myths and misconceptions about the value of QR in health sciences, building on insights from research, teaching experiences, and theoretical perspectives. The goal is not to establish a hierarchy of methodologies but to affirm the importance of qualitative approaches in the health field. It will explore QR ethical and methodological complexities in public health research, higher education practices, and the emerging use of generative artificial intelligence (AI) in this methodology.

## 2 “Despite the low sample size, this qualitative study would benefit from a quantitative analysis (e.g., percentages):” a long journey toward the proper evaluation of qualitative research

Imagine a researcher developing complex and sophisticated statistical procedures without advanced training or experience; now, imagine a peer review process of a quantitative article where the reviewers suggest incorporating qualitative analysis alongside the numbers. For some, this may seem anecdotal, far-fetched, or nearly unbelievable; for others, it is a routine part of their work^1^.

It is important to recognize that incorporating elements—such as frequency counts, or percentages—into qualitative research can misalign with its epistemological foundations. While often well-intentioned, this practice tends to impose positivist standards of rigor on interpretivist paradigms, risking a superficial understanding of the data and undermining the methodological integrity of qualitative research. Such quantification may create an illusion of objectivity or validity, but it detracts from the richness, context, and depth that qualitative approaches aim to preserve. To resist this tendency, several strategies can be adopted: journals should provide clear author and reviewer guidelines that promote adequate reporting of qualitative results; training for editors and reviewers that ensure methodological sensitivity to evaluate these studies; and academic institutions should foster environments that support methodological diversity and integrity. By resisting pressure to conform to quantitative norms, researchers can ensure their studies remain true to qualitative principles while still demonstrating rigor and relevance.

The difficulties in publishing qualitative studies and ensuring adequate review processes in health journals have been widely discussed, revealing rejection policies rooted in positivist perspectives and quantitative lenses that devalue these studies—framing them as lower in priority, citation impact, rigor, value, and interest (1). Condescending discourses frequently describe qualitative papers as interesting, enjoyable, and innovative, yet dismiss them as unsuitable for the scope and standards of health journals or as insufficiently scientific.

The marginalization of health QR is partly related to (1) the relentless search for generalizability, reproducibility, representative samples, and (traditional forms of asserting) objectivity (2); (2) the scarcity of epistemological and practical efforts that recognize the clear role of QR as part of epidemiology and public health, not only in a complementary or secondary role (3); and (3) the lack of knowledge, training and experience on qualitative methodologies. For Banister-Tyrrell and Meiqari there is “a mix of power, inertia and habit, influenced by historical affiliations of epidemiology with positivism and unsatisfactory frameworks for causal inference” that has reinforced suspicions of QR rigor and validity, led to its exclusion from epidemiological studies (3).

There is no shortage of examples that evidence QR relevance in addressing public health challenges. Health research, interventions and policies highly depend on understanding causal relations and social constructs (e.g., wellbeing, access, trust) that require epistemological approaches beyond traditional positivism, recognizing health-related outcomes and people's decision-making as both a biological, socially and culturally embedded experience (3–7). Regarding metrics, qualitative manuscripts were some of the most read, downloaded, and impactful in 2016 (1). In line with other authors, we acknowledge that, like any research method, some qualitative studies may be of poor quality, not well written, inaccessible, or irrelevant to some readers. Moreover, many researchers, editors, reviewers, and readers may lack the interest or training to understand, conduct, or evaluate QR (1). The review of qualitative articles isn't limited to rejection based on quantitative criteria. Articles may also be rejected due to editors' limited familiarity with these methodologies or published without rigorous assessment. It's common to find studies presenting the analysis of a single open-ended question as QR that lack the necessary methodological depth. Instead of focusing on epistemological tensions or the superiority of one approach, efforts should strengthen QR knowledge and skills, promote mixed methods approaches, and endorse higher-quality studies.

The growing number of funding calls that value the use of QR, often rooted in the citizen science paradigm, combined with prejudices that classify QR as “easy,”^2^ and “common sense,” calls for a need to broaden the understanding of phenomena and deepen the multidisciplinary potential of scientific efforts. Additionally, it requires responsible, committed, and ethical research to prevent its potential distortion and ensure the results' quality. High-quality qualitative data, whether from narrative research, phenomenology, grounded theory, ethnography, or case studies (8) (and not solely from interviews, as is often misconceived), emerge only through systematic, thoughtful, and rigorous design and analysis (2). A wide range of methodological protocols has been developed and should be considered to ensure validity and rigor (e.g., triangulation strategies).

QR is resource-intensive, time-consuming, and ethically complex. Budget and time constraints often lead to methodological shortcuts, compromising quality (9). Addressing these challenges requires well-trained teams capable of applying diverse methods across varied contexts and populations.

## 3 Spread the word, break the chain: the possibilities and benefits of integrating qualitative methods into health curricula

Training contexts inevitably reflect the reproduction of a research tradition. In health academia, the (erroneous) belief that numerical data inherently ensures objectivity, validity, and reliability remains dominant. As a result, QR is often seen as secondary, unable to match the “hard” evidence provided by statistical analysis. Moreover, quantitative methodologies continue to dominate postgraduate curricula in health courses, with extensive mandatory training in statistical principles and techniques. In contrast, qualitative methodologies are typically offered as optional courses with a much smaller workload. This perpetuates students' unfamiliarity with QR, limits awareness of its potential, and sustains misconceptions, ultimately contributing to the lack of critical mass in these fields.

Furthermore, postgraduate health students are expected to have a quantitative mindset and filter the concepts of QR through the quantitative lens (10–12). In our experience teaching qualitative methodologies in health courses, feedback from students frequently reflects: (1) a belief that QR is not “real research,” lacks empirical value and is only useful for social research; (2) an acknowledgment of the value of QR, but with a tendency to underestimate the knowledge, skills, and effort required to conduct such studies—reflected in discourses that place qualitative studies as an easier alternative to statistical analysis (13).

Overcoming these myths requires robust curriculum designs integrating qualitative and quantitative paradigms, encouraging students to recognize the limits of positivist approaches and the complementarities rather than the hierarchies of these approaches. Academic institutions should be responsible for including courses that teach students the characteristics, potential, and methodological and ethical complexities of QR. These courses should clearly demonstrate how QR can be effectively applied in public health, serving as key strategies for fostering broader understanding, use and application.

## 4 Ignore, resist, or absorb: the power of reflexivity and deep understanding in a world with (expanding uses of) AI

The emergence of generative AI tools (e.g., natural language processing and machine learning) presents opportunities and challenges for QR (14). AI-driven tools can support time-consuming tasks like transcription and may provide some further support (with caution) in processing textual data, which is especially appealing in an era of increasing demands for speed, competitiveness, and innovation. However, while acknowledging AI's potential, many authors emphasize that human researchers uniquely interpret complex data within the broader context of participants and phenomena (15, 16). Current AI technology cannot grasp meanings in context, the depth of human experiences, social dynamics influence, cultural meanings, or the unique circumstances shaping identified patterns (15, 16).

Integrating AI into qualitative methodologies raises epistemological, methodological, and ethical dilemmas, such as reduced engagement with data, the risk of uniform analysis influenced by software developers, dehumanization, and overreliance on automation—blurring the distinction between systematization and analytical rigor. This may lead to decontextualized, unreflective, and superficial interpretations (6), averaging and erasing often significant singularities. Ethical concerns, including data privacy (17), algorithmic bias (18), and authorship (19), must also be addressed to uphold research quality and academic integrity. These issues are particularly relevant given the critical role of human-driven aspects in QR, such as sensitivity and reflexivity in understanding social and health contexts.

Although researchers have an unquestionable role in interpreting and making meaning from data, under the current conditions of hype, investment and expansion, integrating AI tools feels inevitable. As AI becomes integrated into research processes, establishing standards that uphold the integrity and quality of research becomes increasingly important (20). Equally important is the development of “technological reflexivity,” where researchers (and students) critically understand the implications of using AI-tools (21). Some guidelines for the responsible use of AI in research have already been developed (22) and should be discussed and appropriated.

Moreover, training researchers to use AI tools in ways that support, rather than replace, the essence of qualitative methodology is crucial. For instance, IA can be valuable in automating transcriptions and superficial coding; however, trained researchers must validate its outputs and supplement and further it when relevant, to ensure accuracy and analysis integrity. Notwithstanding the need for further reflection on the role of human cognition in the production of research (and the risks of inverting those who think and those who check), this could support ethical and rigorous forms of integration that help preserve the core strengths of qualitative studies while adapting them to the demands and opportunities of the digital era.

## 5 Discussion

The design of population health policies and practices must be intrinsically linked to understanding individuals, experiences, perceptions, and expectations. Misconceptions about the value and rigor of qualitative methods hinder a comprehensive understanding of public health challenges and limit the development of evidence-based and context-sensitive policies. This, in turn, restricts the potential for more inclusive, equitable, and effective health interventions. Qualitative approaches provide critical insights into the mechanisms behind the success or failure of health interventions and opportunities to enhance their effectiveness.

Deconstructing myths about qualitative data in health requires recognizing the critical role of training and education. Equipping future health research professionals with the knowledge, skills, and tools to apply the most appropriate methodologies must be a priority. By developing curricular structures encompassing diverse methodological approaches and their unique complexities, educational institutions can offer more comprehensive training, fostering well-rounded health researchers and professionals.

The emergence of AI tools presents both opportunities and challenges for QR. While they promise to process large and complex datasets efficiently, they cannot replace human-driven QR interpretative and reflexive nature. Rather than ignoring AI's existence, its strengths should be leveraged to support QR while safeguarding interpretative richness and ethical integrity. This will entail a reflexive and critical understanding of how research processes are being challenged and transformed and developing and adhering to specific guidelines for the responsible use of AI.

If grounded in ethical responsibility, methodological rigor, and quality, qualitative approaches provide critical insights into lived experiences and contextual factors often overlooked. By leveraging these insights, policymakers can design evidence-based, context-sensitive interventions, bridging the gap between research and practice and ensuring more responsive and impactful public health initiatives.

## Footnotes

^1^This section title is a real reviewer comment on a manuscript based on semi-structured interviews.

^2^The terms in quotation marks reflect common remarks frequently encountered by the authors of this article in the academic work.
